# Bridging data and theory: hybrid modelling for pharmaceutical crystallisation process development

**DOI:** 10.1039/d6dd00342g

**Published:** 2026-07-31

**Authors:** Thomas Pickles, Christos Xiouras, Merve Öner, Cameron J. Brown

**Affiliations:** a Strathclyde Institute of Pharmacy and Biomedical Sciences, University of Strathclyde Glasgow G4 0RE UK cameron.brown.100@strath.ac.uk; b CMAC, University of Strathclyde Glasgow G1 1RD UK; c Janssen Pharmaceutica NV Turnhoutseweg 30, 2340 Beerse 2340 Belgium

## Abstract

Crystallisation remains a critical unit operation in pharmaceutical manufacturing, yet process development is often constrained by empirical approaches and data limitations. Mechanistic models such as population balance frameworks provide physical interpretability but require extensive parameterisation and struggle with complex, multiscale phenomena. Data-driven machine learning models offer strong predictive performance but lack transparency, limiting regulatory acceptance. Hybrid modelling, which integrates mechanistic theory with data-driven components, has emerged as a promising strategy to address these limitations. This review surveys the current modelling landscape and critically assesses recent case studies across batch cooling, antisolvent, and continuous crystallisation modes, covering population balance and machine learning combinations. Key open problems are identified, including the absence of agreed modelling and experimental design frameworks, data sparsity, poor scale-up transferability, unresolved regulatory requirements around uncertainty quantification and model. Practical technical recommendations and a proposed workflow are provided to guide future work, with the aim of moving the field beyond isolated case studies toward predictive, explainable, deployable and published crystallisation process development.

## Abstract 2

This perspective reviews the current status, challenges and recommendations for hybrid modelling of pharmaceutical crystallisation whilst offering a framework for wider adoption and publication.

## Graphical abstract

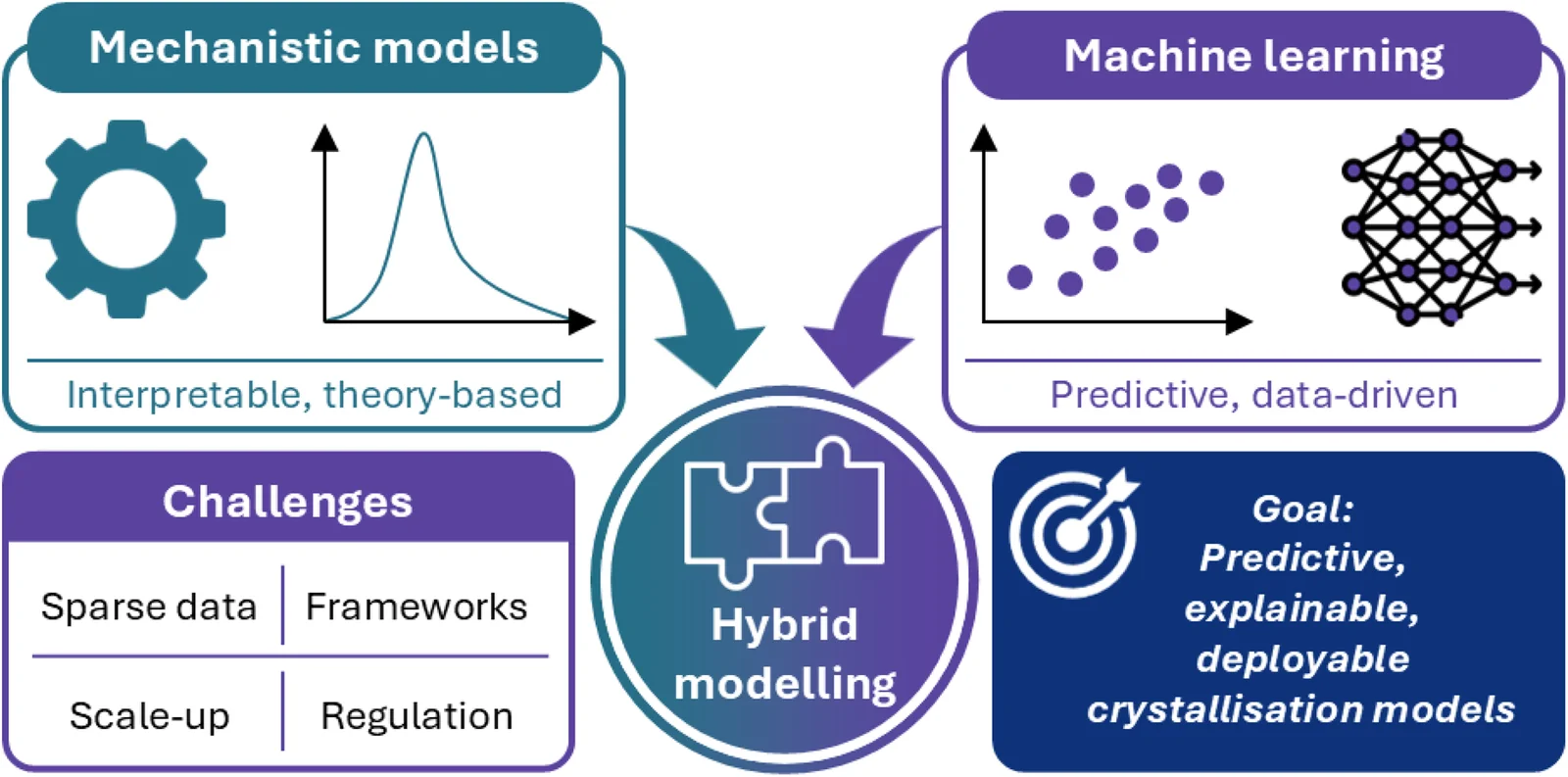

## 1. Introduction

The development of a pharmaceutical molecule into a marketable drug is a resource-intensive and time-consuming process, typically spanning 10 to 15 years and costing an estimated £2 billion.^1^ This process involves numerous stages and multidisciplinary teams, with crystallisation playing a critical role in determining the purity, chemical and physical stability and downstream manufacturability of active pharmaceutical ingredients (APIs).^2,3^

**Fig. 1:**
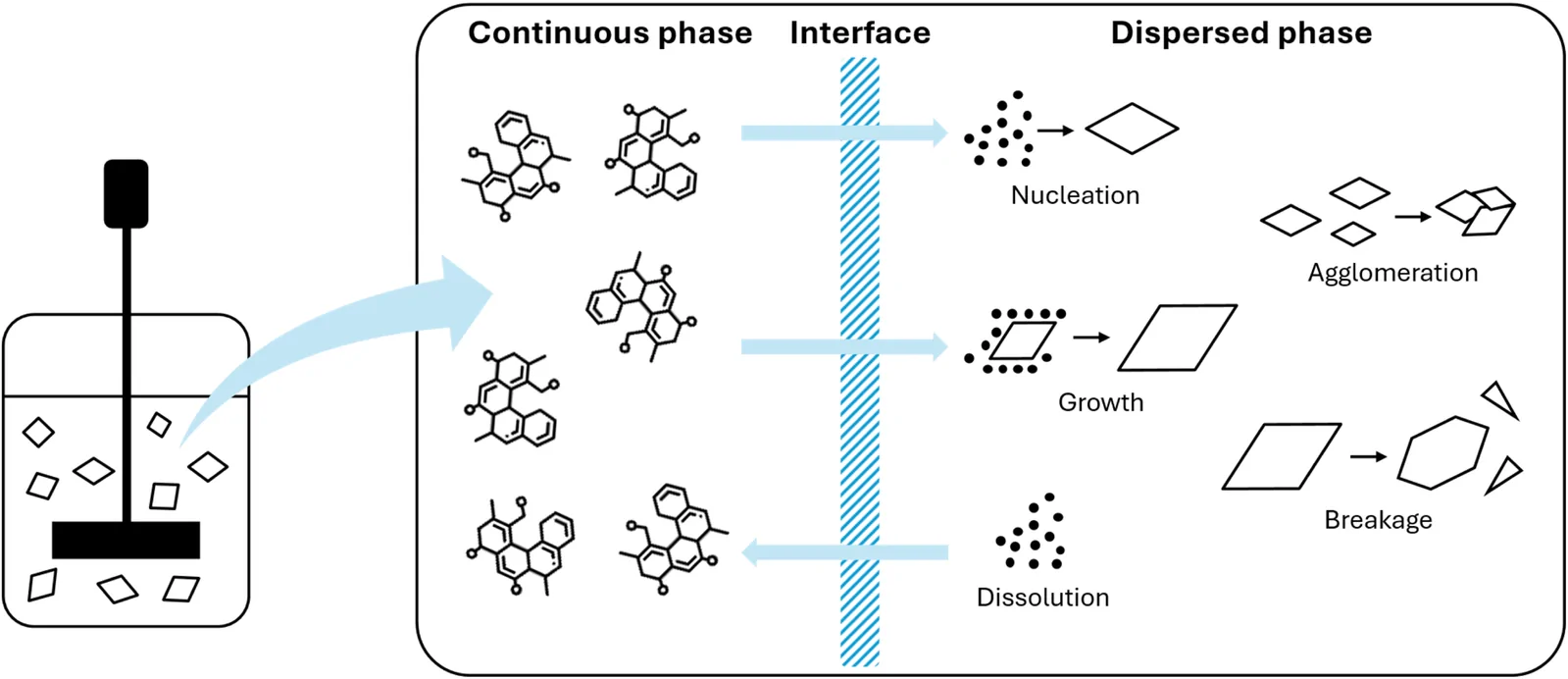
Schematic showing the mechanisms of crystallisation subprocesses. Adapted from Yazdanpanah (2019).^3^

Crystallisation is a solid forming separation and purification process in which dissolved molecules leave a liquid phase and arrange into an ordered crystalline solid. At the process level, it can be understood as the coupled evolution of two phase: a continuous liquid phase, described by properties such as solute concentration and temperature, and a dispersed solid phase, described by the number, size, shape and form of the crystals present. Fig. 1 shows this view of crystallisation as a set of interacting subprocesses: nucleation, growth, dissolution, agglomeration, and breakage. Nucleation and growth transfer material from the continuous phase into the crystal population, dissolution transfer material back to the continuous phase, while agglomeration and breakage redistribute material within the solid phase itself.

**Fig. 2:**
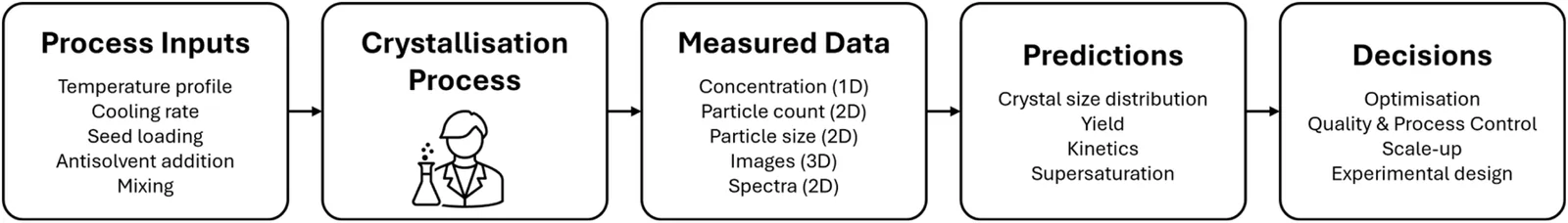
Schematic showing where modelling fits into crystallisation.

Consequently, process optimisation is often inefficient and labour-intensive, with differences in approach (between sites and individuals), formulations and production scales reduce consistency and reproducibility.^4,5^ In response, there is growing interest in model-informed strategies that provide more systematic and predictive frameworks.^6^ Model-informed approaches have demonstrated potential to accelerate development timelines by up to 10 months and reduce clinical trial costs by millions,^7^ and extending such methods to crystallisation (Fig. 2) could reduce the number of laboratory experiments required to identify suitable operating conditions. Thus, accelerating optimisation of cooling, antisolvent and seeding strategies and improving process robustness by identifying operating regions less sensitive to disturbances such as raw material variability, mixing differences or scale-dependent hydrodynamics.^8^

Crystallisation does not, however, conform to a standard modelling problem.^9^ The process is governed by multiscale physics spanning molecular nucleation, particle population dynamics, and reactor-scale transport, with multiple mechanisms (Fig. 1) including nucleation (formation of new particles), growth (increase in crystal size), dissolution, agglomeration (particles sticking together), and attrition (particle breakage) occurring simultaneously and interacting non-linearly.^2,3,10^ Outcomes are highly sensitive to supersaturation (thermodynamic driving force), temperature, mixing, and trace impurities, producing non-stationary behaviour that evolves across conditions and scales, particularly during scale-up where hydrodynamics and impurity profiles change substantially.^11^ From a machine learning perspective, crystallisation deviates from standard problem settings in several compounding respects: data are sparse, noisy, and expensive to obtain; key mechanisms are only partially quantified due to limitations in process analytical technology (PAT);^12–14^ and the system is characterised by hidden states. Datasets are furthermore non-independent and non-identically distributed (non-iid††Non-IID data arises when observations are not independent of one another or when their underlying distributions differ across the dataset. Such conditions violate standard statistical assumptions and can complicate machine learning and statistical analysis.),^15,16^ reflecting time-dependent trajectories, shifting operating regimes, and scale effects. These features collectively limit the applicability and generalisability of purely data-driven models, which often lack the physical interpretability required for reliable process development.^17^

Mechanistic approaches, such as population balance models (PBMs), encode physicochemical knowledge but require extensive parameterisation (where identifiability of parameters can be poor sometimes) and high-quality data for calibration. Despite decades of development,^18^ industrial adoption remains limited due to long development times, system-specific calibration requirements, and uncertainty under real process conditions.^19^ Experimental constraints, including limited resolution of particle size measurements and difficulties integrating high-dimensional PAT data, further restrict model fidelity.^12–14^

Hybrid modelling,^20^ which integrates mechanistic structure with data-driven learning, provides a natural framework for this setting.^21^ By combining physical constraints with statistical flexibility, hybrid models are well suited to multiscale, partially observed systems with sparse, non-iid data. Their adoption nonetheless remains limited by the interdisciplinary expertise required to develop and validate such approaches effectively.^22^ This article reviews the emerging application of hybrid modelling in pharmaceutical crystallisation process development, providing a critical assessment of current modelling approaches, the rationale for hybrid methods, and the practical utility demonstrated in recent case studies.

## 2. Current modelling landscape

Process models are broadly categorised as white-box, black-box, or grey-box based on their transparency and interpretability. White-box models are grounded in theoretical principles and provide clear relationships between inputs and outputs, whereas black-box models rely on data-driven pattern recognition with limited interpretability. Hybrid (grey-box) models aim to balance these trade-offs, improving accuracy and generalisability.

### 2.1 Mechanistic models

**Fig. 3:**
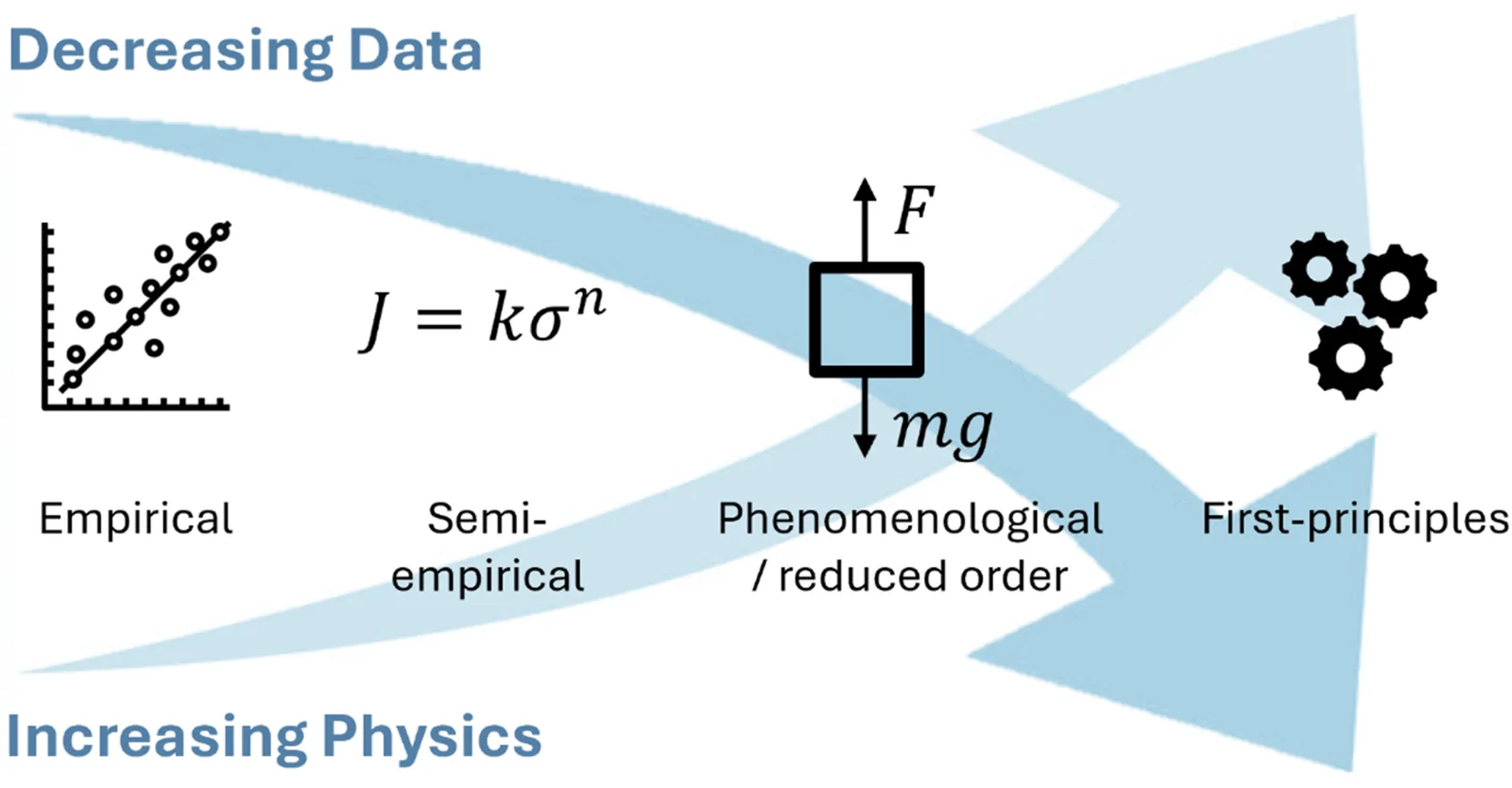
Overview of mechanistic models embedded in process modelling where the models are ordered by their reliance on data and grounding in physics.

Mechanistic models are based on first principles and explicitly defined equations, enabling clear interpretation of process behaviour (Fig. 3). In crystallisation, they span multiple scales:

• Solubility models use empirical, semi-empirical, and mechanistic approaches.^23^

• Kinetic models describe nucleation and growth using theories such as classical nucleation theory^24^ and Burton–Cabrera–Frank,^25^ alongside empirical laws.

• Population balance models (PBMs) are the dominant modelling framework used in crystallisation because they explicitly represent the evolution of particle populations through mechanisms such as nucleation, growth, agglomeration and breakage. They are solved using methods such as moment approaches,^18,26^ method of characteristics^27,28^ or finite volume techniques.^29–31^

• Computational fluid dynamics (CFD) models fluid flow *via* conservation equations.^32^

• Discrete element method (DEM) models particle interactions using Newtonian mechanics.^33^

### 2.2 Data-driven models

**Fig. 4:**
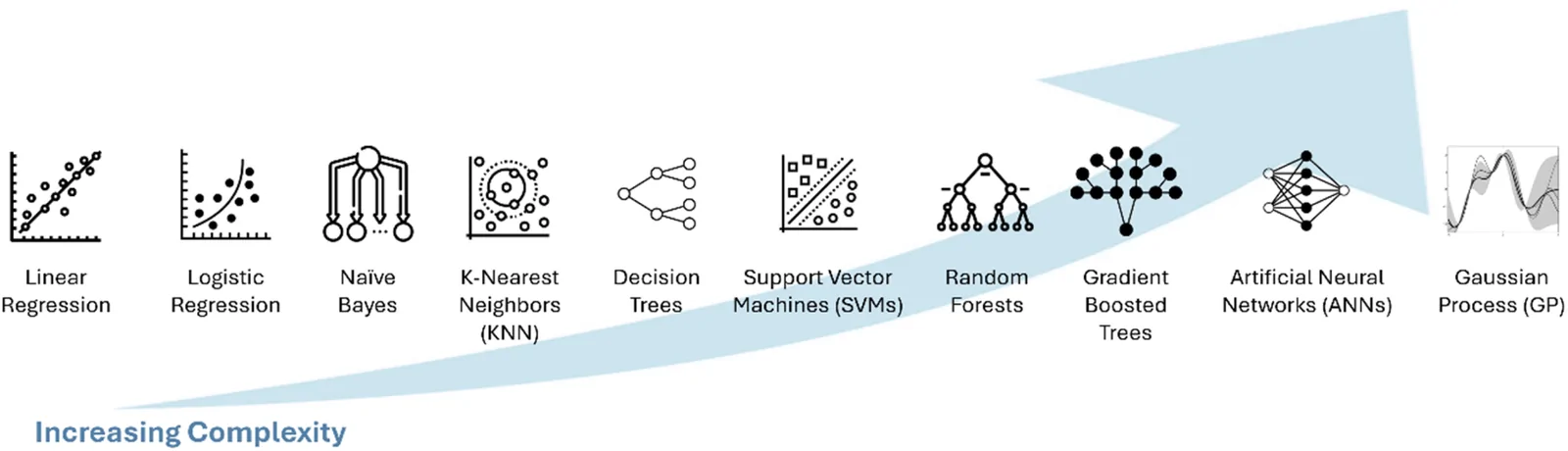
Overview of data-driven models embedded in process modelling where the models are ordered by their estimated ability to learn complex dependencies and computational complexity.^38–40^

Data-driven models vary in interpretability (Fig. 4). Simpler methods (*e.g.* linear regression, decision trees) are interpretable, while complex machine learning (ML) methods capture nonlinear relationships with less transparency. Key approaches include:

• Supervised learning (*e.g.* neural networks, random forests, support vector machines (SVMs)).^34,35^

• Unsupervised learning, including deep learning.^36^

• Statistical methods, such as response surface methodology (RSM) and Bayesian models.^37^

### 2.3 Hybrid models

**Fig. 5:**
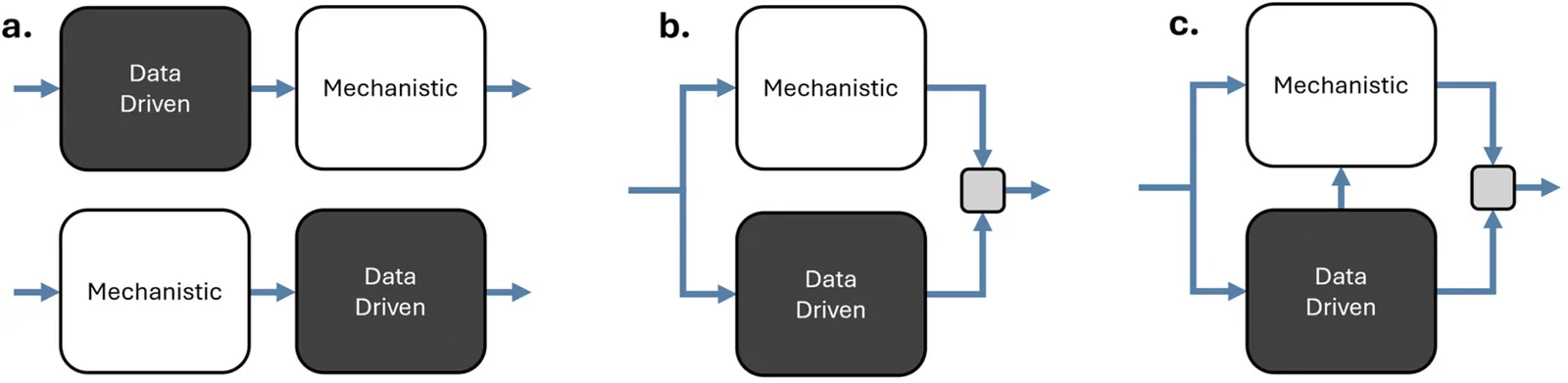
Schematic representation of (a) the two possible serial structure, (b) parallel structure and (c) nested structure for hybrid modelling. For the sake of this article, it is assumed that all data-driven models are black box as algorithms as linear regression are often too simple for pharmaceutical development.

Hybrid models combine mechanistic and data-driven approaches, improving both accuracy and interpretability. They may be sequential (Fig. 5a), parallel (Fig. 5b), or nested (Fig. 5c), with sequential approaches often used in data-scarce settings, *e.g.*, pharmaceutical development, where mechanistic models generate synthetic data for ML training.^20^

## 3. What actually works today

**Fig. 6:**
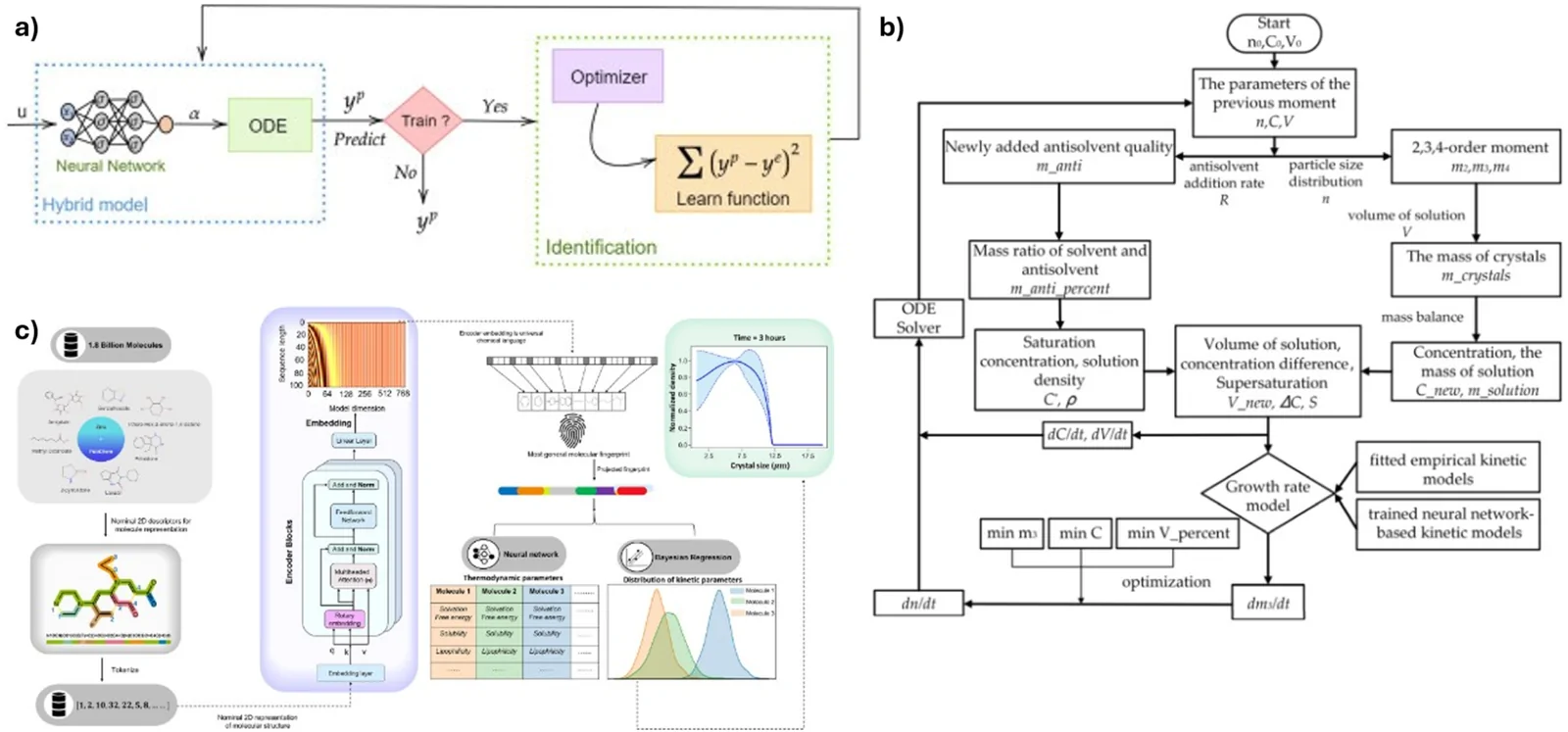
(a) Methodology of UDE employing ANN,^62^ reprinted from *Chem. Eng. Res. Des.*, **200**, 538–549, Copyright 2023, with permission from Elsevier, (b) flowchart of the kinetic simulation,^63^ (c) illustration of the overall CrystalFormer framework, that is developed for relating molecular structure to their process-level performance index like crystal size distribution (CSD).^64^

Outside of crystallisation, hybrid modelling approaches have proven effective in enhancing processes in the chemical,^41–47^ food^44,48,49^ and energy^50^ sectors. These case studies underscore the effectiveness of hybrid approaches in bridging the gap between data-sparse methods and the need for human-understandable pharmaceutical processes. As such, hybrid models have also shown promise in pharmaceutical development, particularly in areas such as synthesis,^51^ solubility prediction,^23,52–54^ material and structure characterisation^55,56^ and drug product formulation.^57–61^Fig. 6 highlights some of the recent implementations of hybrid models in pharmaceutical development.

### 3.1 Batch cooling crystallisation

Batch cooling crystallisation is the most extensively studied crystallisation operation in pharmaceutical development. In this process, a dissolved API is cooled to generate supersaturation, the driving force for the formation and growth of crystals. The principal modelling challenge is predicting how the crystal size distribution evolves during cooling, as this strongly influences filtration performance, downstream processing and product quality. Across most hybrid modelling studies, a population balance model (PBM) provides the mathematical framework used to track crystal populations, whilst machine learning components are used to represent kinetic phenomena such as nucleation and crystal growth that are difficult to measure or model directly.

Lima *et al.*^62^ (Fig. 6a) demonstrated one of the more data-efficient implementations using Universal Differential Equations (UDEs), in which a neural network replaced supersaturation-dependent nucleation and growth terms within a PBM for potassium sulfate crystallisation. With as few as seven experimental batches supplying moment data, concentration, and temperature profiles, the UDE-PBM achieved mean squared and mean absolute errors comparable to conventional PBMs, with particular gains in nucleation prediction accuracy. Learned kinetic terms remained physically interpretable and no full crystal size distribution (CSD) measurement was required. The primary limitation was computational: UDE integration during training was slower than purely data-driven alternatives, and no uncertainty quantification was reported.

Wu *et al.*^65^ pursued a physics-informed recurrent neural network (PIRNN) for aspirin crystallisation, showing that a partial PIRNN requiring only concentration and temperature trajectories (approximately 10^4^ to 10^5^ labelled samples) achieved unmeasured CSD model predictive control (MPC) performance comparable to a full PBM-based controller, against approximately 10^6^ samples required by a purely data-driven recurrent neural network (RNN). The critical limitation is that the study was conducted entirely *in silico*, with the PBM serving simultaneously as the true plant and the source of training physics, a circularity that is unresolved and unrealistic in practice.

Zheng *et al.*^66^ used a validated PBM to generate approximately 1800 open-loop simulated trajectories (∼54 000 samples) for fesoterodine fumarate crystallisation, training RNN and autoencoder-RNN (AERNN) surrogates that reduced CSD MPC solve times by several orders of magnitude and enabled explicit optimisation over individual CSD size classes. The framework is fully contingent on a pre-validated PBM and is unsuitable where kinetics are poorly characterised due to being difficult to estimate experimentally.

Lima *et al.*^67^ demonstrated echo state network (ESN)-based nonlinear supersaturation model predictive control (NMPC) for potassium sulfate crystallisation using a co-teaching dataset combining approximately ten experimental batches with PBM-generated simulations, with the ESN outperforming the multilayer perceptron (MLP) and the long short-term memory (LSTM) architectures for long-horizon supersaturation tracking. Experimental validation was included, though CSD control was not demonstrated and scalability beyond lab scale was not assessed. Lima *et al.*^68^ subsequently trained MLP, RNN, gated recurrent unit (GRU), and LSTM inverse mass and CSD model controllers on approximately 150 NMPC-simulated paracetamol batches (∼30 000 samples), finding the MLP most robust to model mismatch and noise, with lower computational cost than the reference NMPC. Both studies remain simulation-dependent and require retraining under changed process conditions.

### 3.2 Continuous mixed-suspension mixed-product removal (MSMPR) crystallisation

Tadepalli *et al.*^69^ applied multi-objective Bayesian optimisation (MOBO) with Gaussian process surrogates to a three-stage MSMPR cascade for *ortho*-aminobenzoic acid, achieving Pareto fronts of comparable quality to evolutionary algorithms using only ∼140 PBM evaluations *versus* ∼6000. The approach is strictly an offline design tool: no dynamic surrogate was constructed, no closed-loop control demonstrated, and optimal points were not experimentally confirmed.

### 3.3 Antisolvent crystallisation

Dong *et al.*^63^ replaced empirical nucleation and growth kinetics with a neural network for benzophenone antisolvent crystallisation (Fig. 6b), trained on 20 to 26 experimental runs using ATR-FTIR and online imaging data. Coupled with a PBM, this reduced predicted CSD deviation from 5.29% (traditional empirical models) to 1.1% from experimental CSD, with a consistent ∼50% improvement in *R*^2^ compared to empirical kinetic models. The approach requires substantial PAT infrastructure and extrapolation beyond the training domain remains uncharacterised.

### 3.4 Molecular structure-informed and *in silico* frameworks

Pahari *et al.*^64^ proposed CrystalFormer (Fig. 6c), a transformer pre-trained on 1.8 billion molecules, generating SMILES-derived molecular fingerprints to predict solubility and kinetic parameters for input into population balance equations. Thermodynamic and kinetic parameter errors were below 8% for paracetamol and salicylic acid. Validation is restricted to two benchmark active pharmaceutical ingredients (APIs) and the framework is intended for early-stage design rather than real-time deployment.

Kovacs *et al.*^70^ combined PBM-based models with approximately 1300 global multi-objective optimisations for enantiomer second-order asymmetric transformation (SOAT) crystallisation, training random forest classifiers (AUC > 0.85) and regression models (Pearson *r* > 0.95) to predict technology decisions between two competing technologies (with/without wet milling and temperature cycling) and product properties from a synthetic dataset. The framework is entirely *in silico* and requires accurate kinetic models as a prerequisite.

### 3.5 General frameworks

Nielsen *et al.*^71^ proposed a sensor-driven hybrid framework in which a deep neural network (NN) soft-sensor learns nucleation, growth, agglomeration, and breakage kinetics from online particle size data, constrained within a PBM. Validated across laboratory lactose crystallisation, silica flocculation, and an industrial pharmaceutical crystallisation case, this is the most industrially diverse validation set reviewed. A minimum of two to five batches at 3 to 10 minutes measurement intervals was sufficient, and the framework supports online retraining. The inverse problem of inferring unique kinetics from sensor data remains ill-posed.

Ali *et al.*^72^ applied physics-informed neural networks (PINNs) to approximate PBM solutions, demonstrating agreement with exact analytical solutions for size-independent and size-dependent batch growth without terms that may cause numerical diffusion. The study is simulation-only and limited to forward solution of the population balance equation, with no control or optimisation integration.

### 3.6 Summary

The most credible demonstrations aimed at hybrid crystallization process modelling and process control (parameters such as supersaturation and objectives such as CSD) across all the literature found (Table 1), couple a mechanistic PBM with a learned kinetic component rather than replacing the PBM entirely. Experimental validation remains the field's principal weakness: most control-focused studies are simulation-only, and using a PBM as both the training physics and surrogate plant is a circularity that limits transferability claims even if random noise is applied to the simulated data. Data requirements differ markedly by architecture; UDE and PIRNN approaches were effective with fewer than ten experimental batches, whereas RNN surrogates required tens of thousands of simulated trajectories from a trusted first-principles model. No study reviewed has demonstrated closed-loop hybrid control on industrial-scale equipment with full experimental validation, and this remains the outstanding barrier to deployment.

**Table 1:** Literature sources and models used

Name	Year	Paper	Models
Nielsen	2020	Hybrid machine learning assisted modelling framework for particle processes	Data driven – deep neural network (soft sensor)
			Mechanistic – PBM
Lima	2022	Development of a recurrent neural networks-based NMPC for controlling the concentration of a crystallization process	Data driven – RNN
			Mechanistic – PBM embedded in NMPC
Zheng	2022	Machine learning modeling and predictive control of the batch crystallization process	Data driven – RNN and autoencoder–RNN (AERNN)
			Mechanistic – PBM
Wu	2023	Physics-informed machine learning for MPC: application to a batch crystallization process	Data driven – RNN
			Mechanistic – PBM (as a system of ordinary differential equations)
Lima	2023	Improved modeling of crystallization processes by universal differential equations	Data driven – RNN/UDE
			Mechanistic – PBM
Tadepalli	2023	A crystallization case study toward optimization of expensive to evaluate mathematical models using Bayesian approach	Data driven – MOBO (Gaussian process)
			Mechanistic – PBM
Kovacs	2023	A synthetic machine learning framework for complex crystallization processes: the case study of the second-order asymmetric transformation of enantiomers	Data driven – decision tree random forest classifiers
			Mechanistic – PBM
Ma	2024	Digital design of cooling crystallization processes using a machine learning-based strategy	Data driven – NN predictive model
			Mechanistic – mechanistic equations and underlying theory
Lima	2024	Neural-network inverse model controllers for paracetamol unseeded batch cooling crystallization	Data driven – NN inverse model
			Mechanistic – PBM based NLMPC
Dong, Y.	2025	Neural network-based kinetic model for antisolvent crystallization of benzophenone	Data driven – NN
			Mechanistic – PBM
Ali	2025	Data-driven machine learning approach based on physics-informed neural network for PBM	Data driven – PINN
			Mechanistic – PBM
Pahari	2025	Predicting both thermodynamic and kinetic properties of crystallizing molecules *via* transformer-based language model	Data driven – encoder based transformer models and NN
			Mechanistic – PBM

## 4. Open problems

### 4.1 Hybrid modelling framework and model selection

No agreed framework^71^ exists for coupling machine learning and mechanistic components, and structural choices remain largely empirical and compound-specific.^20^ Unlike purely mechanistic or purely data-driven approaches, hybrid models occupy a conceptual grey area: the degree of integration, the partitioning of responsibilities between mechanistic and ML components, and the criteria for selecting one coupling strategy over another are rarely justified systematically in the literature. This absence of unifying theory means that model architectures are frequently tailored to individual projects, limiting transferability and making direct comparison across studies difficult. Establishing principled guidelines for hybrid model construction remains an open and pressing challenge for the field.

### 4.2 Data quality and availability

Hybrid modelling in crystallisation is constrained by the same data limitations that motivate its use. Experimental datasets are typically sparse^73^ and biased towards conditions of practical interest, whilst raw data frequently require substantial preprocessing^74^ due to noise, baseline drift, and outliers common in spectroscopic, imaging, and chord length distribution measurements. Compounding this, crystallisation generates inherently multi-dimensional data^75^ spanning time series, spectral profiles, image-derived distributions, and qualitative process observations, each with distinct structures and error characteristics. The absence of agreed data standards and reporting conventions further limits reuse and aggregation of datasets across studies, restricting model training and benchmarking efforts.

### 4.3 Usability and accessibility

Accessibility in hybrid modelling is best described as a scale determined by the degree of hybridisation. PBMs and classical regression frameworks offer interpretability and established workflows but demand domain expertise and often proprietary software.^76^ More mathematically complex models, *e.g.*, deep neural networks, Bayes statistics or UDEs that can capture highly non-linear phenomena, require considerable data science expertise to fully understand them but are relatively simple to call in *via* Python or web-based graphical user interfaces (GUIs).^77^ A broader cultural challenge therefore persists: there is a need to re-engage researchers with the mechanistic theory underpinning crystallisation, rather than defaulting to black-box ML methods. Addressing this requires better tooling; the field would benefit from reliable, community-maintained packages analogous to PharmaPy^78^ that allow the integration mechanistic solvers with machine learning components *via* standard package managers. Greater choice of well-documented, interoperable tools will lower barriers to adoption and broaden the range of hybrid applications pursued.

### 4.4 Generalisation and extrapolation

Hybrid models trained on laboratory data are susceptible to performance degradation under domain shifts^79^ introduced by impurities, solvent grade variation, or unmodelled/uncertain mechanisms *e.g.*, solubility and supersaturation. Physics-informed architectures partially mitigate this by constraining predictions within physically plausible bounds, but extrapolation reliability remains inferior to purely mechanistic models where kinetics are well characterised. More fundamentally, the well-documented failure of empirical kinetic models to transfer across scales also persists in hybrid frameworks: nucleation and growth parameters identified at laboratory scale frequently do not hold at pilot or manufacturing scale.^80^ No hybrid modelling study reviewed here has demonstrated robust scale-up with experimental validation, and this remains one of the most critical unresolved limitations for industrial deployment. The potential for mechanistic modelling of crystallisation kinetics coupled with ML regression across scales could overcome this.

### 4.5 Regulatory tension

Regulatory expectations increasingly shape the deployment of process models in pharmaceutical manufacturing. Agencies such as the FDA and EMA require that models supporting decision-making are transparent, reproducible, and underpinned by appropriate risk-based justification for their intended use.^81–83^ Recent guidance, including the FDA's draft on Model-Informed Drug Development and ICH M15,^84^ indicates growing openness to hybrid and digital twin frameworks, provided assumptions, limitations, and validation strategies are clearly defined. Practical implementation nonetheless remains challenging: consensus on validating complex hybrid models is limited, and interpretability, traceability of data-driven corrections, and robustness under scale-up or raw material variability remain unresolved.^19^ Standardised frameworks for updating, retraining, and re-qualifying hybrid models are still emerging, and expectations around uncertainty quantification are not consistently defined for systems combining physics-based and machine learning components. Regulatory acceptance beyond established PAT applications therefore remains largely case-dependent, and significant alignment between industrial practice^85^ and regulatory expectations is still required before hybrid modelling can be routinely deployed in crystallisation process development.

### 4.6 Integration with experimental design

Practical deployment of hybrid models in closed-loop experimental and control workflows remains rare. Real-time constraints including computational latency, sensor delay, and the trade-off between model fidelity and convergence speed for parameter estimation present significant barriers to online implementation.^86^ Most hybrid modelling approaches still require human-in-the-loop intervention for model updating, precluding fully automated experimental design. At a higher level, no studies were identified in this review that applied hybrid modelling to model-based design of experiments (MB-DoE),^87^ despite this being a well-established application of purely data-driven approaches.^88,89^ Combining the physical interpretability of hybrid models with the adaptive sampling efficiency of MB-DoE represents a promising and largely unexplored direction and closing this gap should be a priority for future work in crystallisation process development.

## 5. Technical recommendations for hybrid modelling in crystallisation

Future work should prioritise consistent, transparent and scalable practices. To do this we have provided a list of technical recommendations from both the literature and the authors:

• Targeted embedding of ML where mechanistic understanding is weak or absent (*e.g.* rate expressions for nucleation, agglomeration or breakage and effect of impurities on the kinetic mechanisms). The latter case would require use of different input crude material with varying purity profiles or additional impurity spiking experiments to generate comprehensive data.

• Targeted embedding of mechanistic models (*i.e.*, PBM) where data is sparse (*e.g.*, a handful of scaled up process experiments plus a screening dataset of small-scale experiments).

• Physics-informed architectures (UDEs, PINNs) to enforce physical consistency and enhance generalisability.

• Model-based or active experimental design to prioritise data that maximises model learning efficiency with uncertainty quantification through confidence intervals, Bayesian methods or online drift detection.

• Integration of advanced PAT metrics, including image-based particle descriptors, to strengthen model calibration and process observability. Although most commercial image-based PAT vendors do not yet offer off-shelf solutions for advanced image analysis that directly provide particle size and shape measurements, availability of recently published open-source algorithms requiring reduced training effort may help enable this capability.

• Validation across scales, whilst scale-up is still hard to model, to confirm robustness under different hydrodynamic and thermal regimes.

• Utilisation of popular and existing experimental design algorithms *e.g.*, design of experiment (DoE), Bayesian optimisation, genetic algorithms to steer hybrid models towards an experimental integration strategy.

**Fig. 7:**
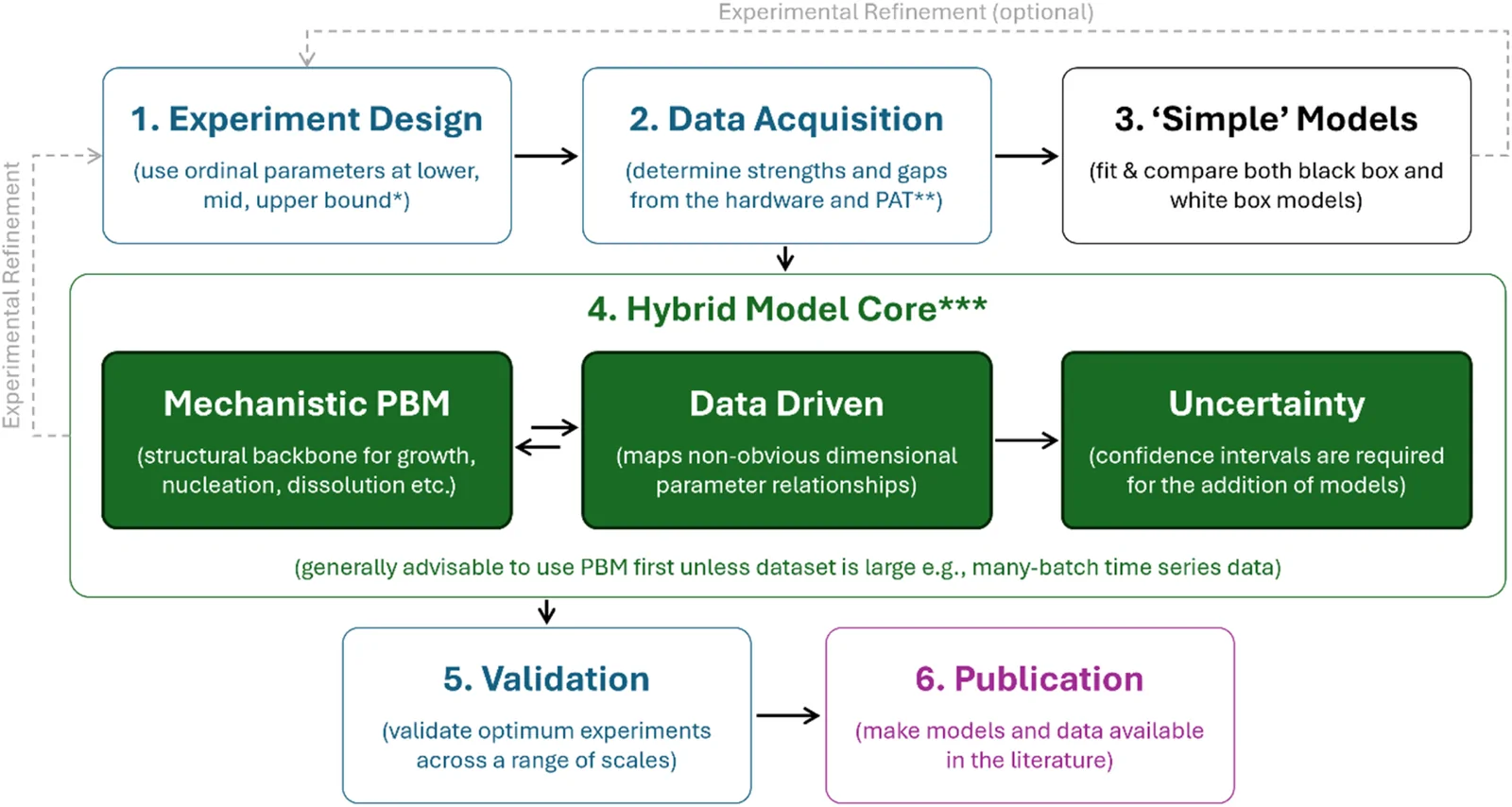
Proposed workflow for integrating hybrid modelling into crystallisation process development and the literature, spanning experimental design, data acquisition, model construction, and multi-scale validation, with optional model comparison between data driven, mechanistic and hybrid models. * A shared design space allows mechanistic and data-driven models to be fitted simultaneously, with parameters adaptable to continuous or mixed strategies as development progresses. ** For example, time-series particle size data may be available without direct kinetic measurements. *** Models can be used to fill data gaps identified in box 2; for example, a PBM-derived mass balance can generate synthetic training data where experimental high performance liquid chromatography (HPLC) data are limited.

Furthermore, we have proposed a simple workflow (Fig. 7) in which the readers can follow to further the literature by demonstrating their own hybrid modelling applications for pharmaceutical crystallisation development and experimental design. The workflow allows users to compare all model types or just progress a purely hybrid modelling approach.

## 6. Conclusion

Hybrid modelling represents a technically credible strategy for pharmaceutical crystallisation process development. By coupling mechanistic population balance models with data-driven components, hybrid approaches address the fundamental limitations of each method in isolation: improving predictive accuracy where first-principles knowledge is incomplete whilst maintaining physical interpretability where data are sparse. Physics-informed architectures such as UDEs and PINNs offer the strongest balance of data efficiency and generalisability, and clear proof-of-concept has been demonstrated across batch cooling, antisolvent, and continuous crystallisation modes.

Nevertheless, the field remains immature. Experimental validation is inconsistent, the majority of control-focused work is simulation-only, and the circularity of using a PBM simultaneously as the source of training physics and a surrogate plant limits transferability claims. Scale-up has not been demonstrated robustly, and integration with closed-loop experimental design and MB-DoE workflows remains largely absent despite representing one of the most practically valuable directions available.

Progress will require standardised modelling frameworks (to further add detail to our from model to publication recommendation), agreed validation criteria, and better open-source tooling to move beyond case-specific demonstrations.^90^ Regulatory alignment on hybrid model qualification and uncertainty quantification remains a prerequisite for routine industrial deployment. The convergence of improved PAT capability, physics-informed machine learning, and model-based experimental design offers a credible path forward. Afterall, the real value of ML in crystallisation is not prediction alone, but guiding experiments under uncertainty.

## Conflicts of interest

Authors Christos Xiouras and Merve Öner are employees of Janssen Pharmaceuticals group of companies.

## Data Availability

No primary research results, software or code have been included and no new data were generated or analysed as part of this review.
